# ROCK Inhibitor Enhances Adhesion and Wound Healing of Human Corneal Endothelial Cells

**DOI:** 10.1371/journal.pone.0062095

**Published:** 2013-04-23

**Authors:** Aurélien Pipparelli, Yvan Arsenijevic, Gilles Thuret, Philippe Gain, Michael Nicolas, François Majo

**Affiliations:** 1 Medicine and Pharmacology, SCGH, University of Western Australia, Crawley, Australia; 2 Unit of Gene Therapy and Stem Cell Biology, Service of Ophthalmology, Jules-Gonin Eye Hospital, University of Lausanne, Lausanne, Switzerland; 3 Laboratory “Biology, Engineering, and Imaging of Corneal Graft”, BiiGC, EA2521, Faculty of Medicine, University of Saint Etienne, Saint Etienne, France; 4 Institut Universitaire de France, Paris, France; 5 Unit of Research on Lens and Cornea, Jules-Gonin Eye Hospital, University of Lausanne, Lausanne, Switzerland; University of Reading, United Kingdom

## Abstract

Maintenance of corneal transparency is crucial for vision and depends mainly on the endothelium, a non-proliferative monolayer of cells covering the inner part of the cornea. When endothelial cell density falls below a critical threshold, the barrier and “pump” functions of the endothelium are compromised which results in corneal oedema and loss of visual acuity. The conventional treatment for such severe disorder is corneal graft. Unfortunately, there is a worldwide shortage of donor corneas, necessitating amelioration of tissue survival and storage after harvesting. Recently it was reported that the ROCK inhibitor Y-27632 promotes adhesion, inhibits apoptosis, increases the number of proliferating monkey corneal endothelial cells *in vitro* and enhance corneal endothelial wound healing both *in vitro* and *in vivo* in animal models. Using organ culture human cornea (N = 34), the effect of ROCK inhibitor was evaluated *in vitro* and *ex vivo*. Toxicity, corneal endothelial cell density, cell proliferation, apoptosis, cell morphometry, adhesion and wound healing process were evaluated by live/dead assay standard cell counting method, EdU labelling, Ki67, Caspase3, Zo-1 and Actin immunostaining. We demonstrated for the first time in human corneal endothelial cells *ex vivo* and *in vitro*, that ROCK inhibitor did not induce any toxicity effect and did not alter cell viability. ROCK inhibitor treatment did not induce human corneal endothelial cells proliferation. However, ROCK inhibitor significantly enhanced adhesion and wound healing. The present study shows that the selective ROCK inhibitor Y-27632 has no effect on human corneal endothelial cells proliferative capacities, but alters cellular behaviours. It induces changes in cell shape, increases cell adhesion and enhances wound healing *ex vivo* and *in vitro*. Its absence of toxicity, as demonstrated herein, is relevant for its use in human therapy.

## Introduction

Preservation of corneal transparency is essential for vision. This requires integrity of the three layers of the cornea, the stratified squamous epithelium, the stroma and the inner surface endothelium. The corneal endothelium is a monolayer of cells, which is formed from neural crest-derived cells. During development, corneal endothelial cells (CEC) proliferate and migrate centrally to form a continuous mosaic of cells, facing the aqueous humor. Cell-cell contact induces growth arrest in G1 phase through contact inhibition mechanism, leading to the formation of a monolayer with a defined endothelial cell density (ECD) [1]. The corneal endothelium is responsible for the passive diffusion of nutriments from the aqueous humor and for the hydration of the cornea through its barrier and ionic pump functions [2]. Several studies have shown that human endothelial cells do not replicate *in vivo*, even if they retain a proliferative potential, as seen in *ex vivo* wound healing experiment or *in vitro* [3], [4]. A recent study demonstrated that a few proliferating cells were found exclusively in extreme periphery of endothelium on human corneas with a short postmortem time and that a very slow and continuous centripetal cell migration might exist to partially compensate the physiological cell loss in vivo [5]. Nevertheless, this mechanism cannot immediately compensate neither acute nor chronic important CEC losses which are replaced by enlargement and migration of neighboring cells resulting in shape modification and increase of cell size [6], [7]. In physiologic conditions the insufficient proliferative capacity leads to a gradual ECD decrease of 0.3–0.6% per year [8]. This decrease can be accelerated as a result of accidental trauma, certain systemic diseases like diabetes [9], treatment for glaucoma [10] or endothelial dystrophies [11]. When ECD falls below a critical threshold, the barrier and “pump” functions of the endothelium are compromised and this results in the formation of a corneal oedema and loss of visual acuity. The conventional treatment for such severe disorder is corneal transplantation, including penetrating keratoplasty and endothelial lamellar graft. Human corneas harvesting, evaluation, preservation and distribution are under the responsibility of eye banks, which stores corneal tissue either for short term at 2–6°C (Cold storage USA and Asia) or for long term at 30–32°C in culture medium (Organ culture, mostly in Europe). Unfortunately, there is a worldwide shortage of donor corneas available for transplantation.

Several approaches have been evaluated to overcome this lack of tissues. Improvement of surgical procedures allows optimizing the use of corneal graft, especially by lamellar technique, according to the principle of split cornea transplantation for two recipients (anterior and posterior) [12]. In order to extend the EC viability of organ-culture cornea, an anti-apoptotic gene therapy was also assessed. Experiments demonstrated that expression of anti-apoptotic proteins Bcl-xL or p35 allowed limiting the cell loss and increasing the number of available donor’s corneas. [13]. During the last decades, in vitro culture technics of human corneal endothelial cells (HCEC) have greatly improved. These cells can be isolated and expanded in culture either as a monolayer [14] or as sphere-forming colonies [15] and a number of studies showed the possibility to transplant them as a cellular sheet with or without a carrier or by injecting them directly into the anterior chamber in animal models [16], [17]. Medical treatments are also considered in order to cure corneal endothelial diseases directly *in vivo*. One promising target is the Rho family GTPase signalling and its best characterised downstream effector, Rho-associated, coiled-coil-forming protein kinase (ROCK). Trough phosphorylation of myosin light chain (MLC), myosin light chain phosphatase (MLPC) and LIM kinase (LIMK), ROCK regulates the formation of actin stress fibers assembly and cell contraction [18]. ROCK controls also, via phosphorylation of Na^+^/H^+^ exchanger 1 (NHE1), the formation of focal adhesion, which linked stress fibers to the extracellular matrix, an important step involved in cell adhesion and motility [19]. In addition to these primary functions in cytoskeleton remodeling and migration, this pathway has been shown to be involved in the regulation of other biological processes like gene transcription, G1 cell cycle progression and apoptosis [20], [21]. ROCK inhibitor molecule seems to be promising for the treatment of a wide-range of pathologies including cancer, neuronal degeneration, kidney failure, asthma, glaucoma, osteoporosis, erectile dysfunction and insulin resistance [22]. In ophthalmology, this inhibitor has been evaluated in corneal endothelial cells using rabbit and monkey animal models. This molecule inhibited dissociation-induced apoptosis and promoted the adhesion and proliferation of monkey corneal endothelial cells (CEC) [23]. Furthermore, ROCK inhibitor is able to increase wound healing process in monkey CEC and *in vivo* by topical treatment of rabbits wounded by transcorneal freezing [24]. Recently, it has been shown that modulation of cell adhesion by ROCK inhibitor allows enhancing EC engraftment in a primate model of endothelial dysfunction [25], leading to the grant of a patent application [26].

Here, we proposed to evaluate the effects of ROCK inhibitor on HCEC *in vitro* and *ex vivo*, firstly to assess the potentiality to increase the number of corneal graft available for eye banks and secondly to further validate the previous results obtained in animal models, a step required before first in man application.

In the present study, we demonstrated for the first time on HCEC that ROCK inhibitor is not toxic, does not induce proliferation and does not modulate apoptosis. However, it promotes corneal endothelial wound healing by enhancing endothelial remodeling, adhesion and cell migration.

## Materials and Methods

### Materials

Serum-free medium (OptiMEM-1), Petri dish, multiwell tissue culture plates, fetal bovine serum (FBS) were purchased from Fisher Scientific (Wohlen, Switzerland). Medium 199 (M199), epidermal growth factor (EGF; from mouse submaxillary glands), nerve growth factor (NGF; from mouse submaxillary glands), bovine pituitary extract (also known as Keratinocyte Growth Supplement), ascorbic acid, calcium chloride, chondroitin sulphate, EDTA (EDTA disodium salt), antibiotic/antimycotic solution, gentamicin, trypsin and ROCK inhibitor (Y-27632) were purchased from Sigma-Aldrich (Buchs, Switzerland). Cell attachment reagent (FNC Coating Mix) was purchased from GENTAUR (Brussels, Belgium). TRIzol Reagent, Oligo(dT)_12–18_ primer, dNTP Mix (10 mM), Ribonuclease H, dithiothreitol (DTT; 0.1 M), 5X First-Strand Buffer, SuperScript™ II Reverse Transcriptase, Click-iT® EdU Cell Proliferation Assays, Zo-1 primary antibody, Alexa Fluor 555/633® and Live/Dead Assay Kit were purchased from Invitrogen (Zug, Switzerland). Taq PCR Master Mix was purchased from Qiagen (Hombrechtikon, Switzerland). ROCK 1 and ROCK 2 forward and reverse primers were purchased from Microsynth (Balgach, Switzerland). Ki67 primary antibody and antifading fluorescent mounting medium were purchased from DakoCytomation (Baar, Switzerland). Caspase3 primary antibody was purchased from BD (Allschwil, Switzerland) and CorneaMax medium from Eurobio (Les Ulys, France).

### Human Corneal Tissue

All procedures conformed to the tenets of the Declaration of Helsinki for biomedical research involving human subjects. All study corneas were received from Lausanne Eye Bank, and had been considered to be unsuitable for transplantation. In accepting corneas from Lausanne Eye Bank, the overall health of the donor before death was considered and tissue was rejected from donors with previous history or treatment that might have damaged the corneal endothelium. Criteria for exclusion were: too long a period between time of death and time of preservation, corneas from donors with diabetes, glaucoma, sepsis, or ocular infection, or from donors who were on large doses of chemotherapeutic agents. After retrieval, corneas were placed in 100 ml of CorneaMax at 32°C in a dry incubator, according to the standard organ-culture (OC) in place in the Lausanne Eye Bank. For the *ex vivo* and *in vitro* studies 17 pairs of OC corneas [mean donor age: 73+/− SEM 3 years (median 73; range 47–91); mean time from death to procurement: 18+/−1 hours (18; 9–27)] and 7 OC corneas [mean donor age: 79+/−4 years (85; 64–86); mean time from death to procurement: 19+/−6 hours (19; 2–40)] were used respectively.

### Primary Cell Culture

HCEC were isolated and cultured according to published protocols [27]. Corneas were removed from the conventional OC medium and washed several times with M199 containing 50 µg/ml gentamicin before being placed in a Petri dish. Descemet’s membrane with intact endothelium was carefully dissected in small strips and then incubated in OptiMEM-I supplemented with 10% FBS overnight to stabilize the cells before culture. After centrifugation, the strips were incubated in 0.02% EDTA solution at 37°C for 1 hour to loosen cell–cell junctions. Cell junctions were disrupted by forcing the tissue and medium multiple times through the narrow opening of a flame-polished pipette. Cells were peeled and re-suspended in High Medium (HCEC conventional proliferative culture medium) containing OptiMEM-I, 10% FBS, 5 ng/ml EGF, 20 ng/ml NGF, 100 µg/ml pituitary extract, 20 µg/ml ascorbic acid, 200 mg/l calcium chloride, 0.08% chondroitin sulphate, 50 µg/ml antibiotic/antimycotic solution diluted 1/100. Isolated cells and pieces of Descemet’s membrane that still contained attached cells were plated in 6-well tissue culture plates that had been precoated with undiluted FNC Coating Mix. Cultures were then incubated at 37°C in a 5% carbon dioxide, humidified atmosphere. High Medium was changed every 2 days. After primary cultures reached confluence, cells were trypsinized, filtered and seeded at the same number per well in a 12 well tissue culture plate and stored at 37°C in High Medium until reach 50% or 100% confluence, depending the experiments. Cells were then extensively washed with PBS and treated with ten µM Y-27632 diluted in High Medium or Low Medium composed of OptiMEM-I plus 4% FBS (mean serum concentration used by Eye Bank in OC medium). Except for ROCK1 and ROCK2 mRNA expression, all experiments were repeated with three different biological samples and performed in triplicates for each condition.

### ROCK 1 and ROCK 2 mRNA Expression in OC and Primary Culture HCEC

#### Ex vivo HCEC isolation

Two pairs of OC cornea were used in order to evaluate the expression of ROCK 1 and ROCK 2 mRNA in HCEC. Under an operating microscope, Descemet’s membrane with endothelium was peeled off from the underlying stroma with forceps to avoid contamination by other cell types. Tissues were then frozen at −80°C until RNA isolation.

#### In vitro HCEC isolation

Confluent cell cultures (P1) were washed twice with PBS and then incubated during 2 days in High or Low Medium. Cells were then trypsinized, pelleted and frozen at −80°C until RNA isolation. Experiment was performed independently with two biological samples.

#### RNA isolation and reverse transcription

Total RNA was isolated from HCEC using the TRIzol solution according to the manufacturer's instructions. First-strand cDNA synthesis was carried out on 1 µg of total RNA in a final volume of 20 µL with SuperScript™ II Reverse Transcriptase as per the manufacturer’s protocol. Briefly, after addition in nuclease-free microcentrifuge tubes of 1 µg of total RNA, 0.1 µL Oligo(dT)_12–18_ (500 µg/ml), 1 µL dNTP Mix (10 mM each) and sterile distilled water to complete the volume at 12 µL, the mixture was heated at 65°C for 5 minutes. 4 µL of 5X First-Strand Buffer and 2 µL of DTT were then added and the mix incubated at 42°C for 2 minutes. Incubation at 42°C for 50 minutes was performed after the addition of 1 µL of SuperScriptTM II RT. The reaction was inactived by heating at 70°C for 15 minutes. To remove RNA complementary to the cDNA, 1 µL of E. coli RNase H (two units) was added and the mixture incubated at 37°C for 20 minutes and then chilled on ice. cDNA were stored at −20°C until use in PCR.

#### PCR

PCRs were performed using 1 µL of RT products, 2.5 units of Taq DNA Polymerase, 1x PCR Buffer (containing 1.5 mM MgCl_2_), 200 µM of each dNTP and 0.5 µM of each primer. The sequences of human ROCK 1, ROCK 2 and GAPDH primers (Yin, 2008, Friel, 2005) were respectively: sense 5′-GAAGAAAGAGAAGCTCGAGA-AGAAGG-3′, antisense 5′-ATCTTGTAGCTCCCGCATCTGT-3′; sense 5′-AATTCACTGTGTTT-CCCTGAAGATA-3′, antisense 5′-TTCATTTTTCCTTGATTGTATGGAA-3′ and sense 5′-TGCACCACCAACTGCTTAGC-3′; antisense 5′-GGCATGGACTGTGGTCATGAG-3′. Amplifications were carried out using the following cycling parameters: initial denaturation at 95°C for 10 minutes, denaturation at 95°C for 60 seconds, annealing at 60°C for 60 seconds, and extension at 72°C for 60 seconds. PCR amplification was done for 32 cycles with a final extension at 72°C for 10 minutes. For negative controls, PCR was run on RT products performed on water and on samples without RT enzyme. The PCR products were subjected to electrophoresis on 1% agarose gels containing SYBR® Safe DNA Gel Stain. Stained gels were captured using a digital camera.

### Toxicity, Viability and Apoptosis Assessment

#### Ex vivo live/dead assay

In order to determine the effect of ROCK inhibitor on HCEC viability, live-dead assays were conducted on three pairs of OC corneas with or without treatment for 48 hours with ten µM Y-27632. The live/dead assay was performed according to published protocols [28]. Briefly, after the 48 hours of incubation corneas were washed in PBS, placed endothelial side up in a sterile Petri dish, incubated for 45 minutes at 37°C with 100 µL of Hoechst 33342 (10 µM), Ethidium Homodimer-I (4 µM) and Calcein-AM (2 µM), and then gently rinsed in PBS. After having done a radial cut on corneas to allow a flat mount without folding, corneas were mounted endothelial side up under a coverslip using an antifading agent. Five tagged image format file images (a central image and one in each quarter) were acquired using a microscope (BX60; Olympus, Tokyo, Japan) with a x4 objective for the Calcein and x10 objective for the Hoechst and Ethidium stainings. Viability, corresponding to the Calcein staining, was measured on the totally of the 5 images using the Cell^∧^P software (Cell^∧^P; Cell Imaging, Hamburg, Germany). After background noise removal, signal thresholding, and binarization, the calcein surface area was automatically calculated, as was the ratio of viable surface area to total analyzed area. In determined Region Of Interest (ROI) of the five images, all the positive Hoechst and Ethidium nuclei were counted. The mortality rate was the number of positive Ethidium nuclei out of the total number of nuclei (Hoechst+Ethidium). Relative ECD in the selected areas corresponded to the ratio between the number of nuclei and the delineated measurement area. The mean of the five images was considered.

#### In vitro live/dead assay

In order to test the effect of Y-27632 treatment on HCEC viability, studies were conducted using P1 confluent and non-confluent HCEC cultures. Cells in a 12-well tissue culture plate were extensively washed with PBS and treated 2 days with ten µM Y-27632 diluted in High or Low Medium. The cell-viability assay, as described previously, was performed to determine overall viability. Three randomly selected images per well were acquired using a microscope with a x10 objective for the three staining. Mortality, Relative ECD and Viability were determined as described previously. Cells fixed in absolute methanol for 10 minutes at −20°C before staining were used as positive control. All experiments were repeated with three different biological samples and performed in triplicates for each condition.

#### Ex vivo and in vitro apoptosis assessment

In order to determine the involvement of Y-27632 on HCEC apoptosis, Caspase3 immunostaining was performed on three pairs of OC corneas after treatment with ROCK inhibitor. Briefly, corneas were fixed with 4% PFA, blocked and permeabilized in 3% BSA, 1% Triton X-100. Corneas were incubated with Caspase3 primary antibody (1∶50) at RT for 2 hours and then incubated with a 1∶500 dilution of the Alexa Fluor 633®-conjugated secondary antibody. To assess specificity of the immunostaining, corneas were processed without primary antibody. Nuclei were counterstained with DAPI and corneas flat mounted side up under a coverslip using an antifading mounting medium. The apoptosis assessment was also performed on confluent primary cell cultures (P1) after Y-27632 treatment. Cells were subcultured in High Medium until reached 100% confluence. Cells were then extensively washed with PBS and treated 2 days with ten µM Y-27632 diluted in High Medium or Low Medium. Caspase3 immunolocalization in cultured cells was performed with the same protocol as described above. Staining was visualized using a fluorescence microscope.

### Proliferation Assessment

#### Ex vivo

Proliferation assessment was evaluated by three methods: (1) Routine CEC count usually performed in the Lausanne Eye Bank (2) Click-iT® EdU Cell Proliferation Assays (3) Ki67 immunostaining, which has been proven as a reliable proliferation cell marker for *ex vivo* and *in vitro* HCEC [29]. Six pairs of OC corneas were used for the proliferation assessment.

#### Standard endothelial cell count

After washing in BSS, corneas were placed endothelial side up in a sterile Petri dish. Dead cells were identified using 0.4% trypan blue only, to eliminate corneas with extensive CEC necrosis. The endothelial surface was incubated with 0.9% sodium chloride for 4 minutes to dilate the intercellular spaces. Once the cell contours were optimally discernible, the endothelium was viewed through a long working distance x10 objective using a light direct microscope and endothelial photographs were acquired. ECD was determined manually on three randomly chosen non-adjacent zones of the endothelium contained within the central 8 mm diameter. This ECD evaluation was performed three times: (1) just after corneas procurement (2) just before ten µM Y-27632 addition in OC medium after a mean storage time of 14+/−2 days and finally (3) after a supplemental time of conservation of 13+/−3 days.

#### Click-iT® EdU cell proliferation assays

Ten µM EdU was added to the medium at the same time as Y-27632, detection was performed. HCEC that had integrated EdU during S phase were detected as follows. Briefly, Corneas were washed twice with PBS and fixed with 4% PFA at room temperature (RT) for 30 min. Following fixation, corneas were washed twice with 3% BSA and treated with 1% Triton X-100 at room temperature for 20 min. After permeabilization, corneas were washed twice with 3% BSA. The corneas, placed endothelial side up in Petri dish, were incubated at room temperature in the dark for 30 min with 100 µL Click-iT reaction mixture (1× Click-iT reaction buffer, CuSO4, Alexa Fluor 488®-azide, and reaction buffer additive). Corneas were washed once with 3% BSA and twice with PBS. Nuclei were counterstained with DAPI and corneas flat mounted under a coverslip using an antifading agent. Observation was performed under fluorescence microscope (BX60; Olympus, Tokyo, Japan). Five images (a central and one in each quarter) were acquired with a x10 objective. The mean of the five images was considered.

#### Ki67 immunostaining

Three of the six corneas pairs used previously for the EdU assay were evaluated. After EdU revelation, corneas were washed three times with PBS and incubated in 3% BSA, 1% Triton X-100 in PBS for 30 min at RT in order to reduce nonspecific background staining and to permeabilize cells. Corneas were then incubated with Ki67 primary antibody (1∶100) at RT for 2 hours. Following primary antibody incubation, corneas were washed with PBS, incubated for 10 min with blocking buffer, and then incubated for 1 hour with a 1∶500 dilution of the Alexa Fluor 633®-conjugated secondary antibody. Corneas were mounted endothelial side up under a coverslip using an antifading agent after counterstaining of the nuclei with DAPI. Positive staining was visualized using the same fluorescence microscope. To assess specificity of the staining, corneas were processed without primary antibody.

#### In vitro

Treatment conditions included the following: (1) incubation of cells in High Medium without Y-27632; (2) incubation of cells in High Medium with Y-27632; (3) incubation of cells in Low Medium without Y-27632, or (4) incubation of cells in Low Medium with Y-27632. After primary cultures reached confluence, cells were trypsinized, filtered and seeded at the same number per well in a 12-well tissue culture plate and incubated 24 hours at 37°C in High Medium to ensure attachment to the matrix. Cells were further extensively washed with PBS and treated with ten µM Y-27632 diluted in High or Low Medium supplemented with ten µM EdU until confluence. Medium was changed every 2 days. HCEC proliferation kinetic was observed every day until cells reach confluence under microscope. Proliferation was assessed by Click-iT® EdU Cell Proliferation Assays and by Ki67 immunostaining as describe previously for the *ex vivo* proliferation evaluation. Proliferation was also assessed, *in vitro* by the determination of the relative ECD. In determined region of interest (ROI) of the five images, all the positive EdU cells and DAPI nuclei were counted. The % of EdU^+^ cells rate was the number of positive EdU cells out of the total number of nuclei. The mean of the five images was considered. Observation was performed with a microscope (Axiovert 200; Zeiss, Oberkochen, Germany).

### Morphological Assessment

HCEC morphology was evaluated on *ex vivo* corneas and *in vitro* after Y-27632 treatment using zona occludens and actin staining with respectively Zo-1 antibody and phalloidin. Briefly, corneas and confluent cells culture (P1) were fixed with 4% PFA, blocked and permeabilized in 3% BSA 1% Triton X-100. Corneas and HCEC culture were incubated with Zo-1 primary antibody (1∶100) at RT for 2 hours, washed with PBS and incubated with a 1∶500 dilution of the Alexa Fluor 488® or 633®-conjugated secondary antibodies. For actin staining, corneas and cell cultures were incubated 1 hour at RT with a 1∶200-dilution Alexa Fluor 488® phalloidin in the dark. Nuclei were counterstained with DAPI and corneas flat mounted side up under a coverslip using an antifading. Staining was visualized using a fluorescence microscope for the *ex vivo* samples and for cell cultures.

### Wound Healing Assessment

#### Ex vivo

A calibrated endothelial wound, using a 10 µL plastic pipette tip, was performed on whole corneas center (n = 3 pairs) and ten µM Y-27632 was added to the OC medium of one cornea of each pair. For detection of proliferation, ten µM EdU was added in OC medium at the same time than Y-27632. Corneas were then stored at 32°C in a dry incubator. Every day wound healing was observed after corneas incubation with 0.9% sodium chloride under light microscope until closure. Alizarin red staining was performed on corneas after totally wound closure of the first cornea, either control or treated samples, and HCEC observed under microscope.

#### In vitro

HCEC were cultured at confluence in 12-well culture plates in High Medium (P1). Cells were washed in PBS and scraped with a 10 µL plastic pipette tip. Cells in suspension were washed off with PBS. The culture medium was then replaced with High or Low Medium containing ten µM Y-27632 and ten µM EdU. The wound distance, that is, the distance between the cells existing at one edge of the linear defect and those existing at the opposite edge of the defect, was then determined by use of Cell^∧^P software after 0, 2, 4, 6, 8, 10, 12 and 24 hours of incubation. Proliferation and relative ECD was also assessed by respectively the % of EdU positive cells and the number of DAPI positive nuclei per region of interest as described previously.

### Cell Adhesion Assay

The adhesion of HCEC to FNC was tested as described elsewhere [30]. Confluent primary cultured cells (P0) were washed twice with PBS and then pretreated 30 min with High Medium or Low Medium supplemented or not with ten µM Y-27632. Cells were trypsinized, resuspended in their corresponding culture medium, filtered and adjusted to an equal cell number of 2*10^5^/ml. Fifty µl of cell suspension was added per well into a 96-well plates that had been precoated. Cells were allowed to adhere to the matrix for 2 or 6 hours; nonadherent cells were then washed off with PBS. Cells were fixed with 4% paraformaldehyde for 10 minutes at RT and stained with 0.1% crystal violet solution for 60 min. Plates were extensively washed with water to remove excessive staining, and the dye was solubilized with 10% acetic acid. Absorbance at 570 nm was quantified on a microplate reader (MRX II; Dynex Technologies). Adhesion morphology was also assessed on HCEC cultures by actin staining. As described earlier, cells treated or not with Y-27632 were allowed to adhere to the FNC coating for 6 hours and nonadherent cells were then washed off with PBS. Actin was directly stained with Alexa Fluor 488® phalloidin and cells observed under fluorescence microscope as described previously.

### Statistical Analysis

All data are presented as the mean +/− SEM. Statistical significance was determined with Student’s t test or with two way Anova test. P<0.05 indicated a significant difference.

## Results

### Presence of ROCK 1 and ROCK 2 Transcripts in HCEC Ex vivo and In vitro

**Figure 1:**
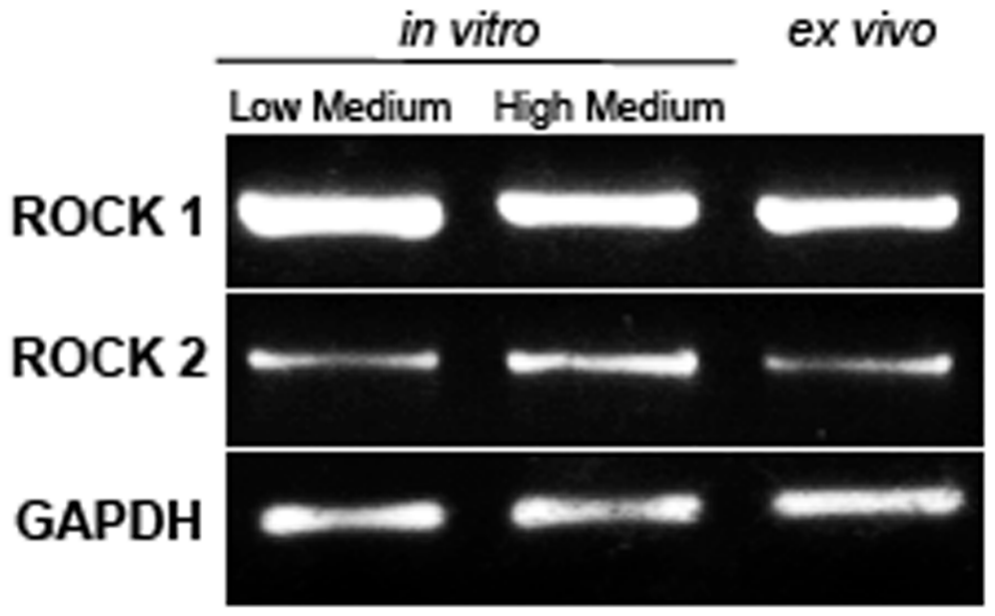
mRNA expression of ROCK 1–2 in HCEC *ex vivo* and *in vitro*. Rho kinase (ROCK) 1 and ROCK 2 mRNA are expressed in human corneal endothelial cells from *ex vivo* corneas and primary cell cultures in Low as High Medium.

As shown in Figure 1, the mRNAs for ROCK 1 and 2 were detected in HCEC in all tested conditions.

### Ex vivo Toxicity, Viability and Apoptosis Assessment

**Figure 2:**
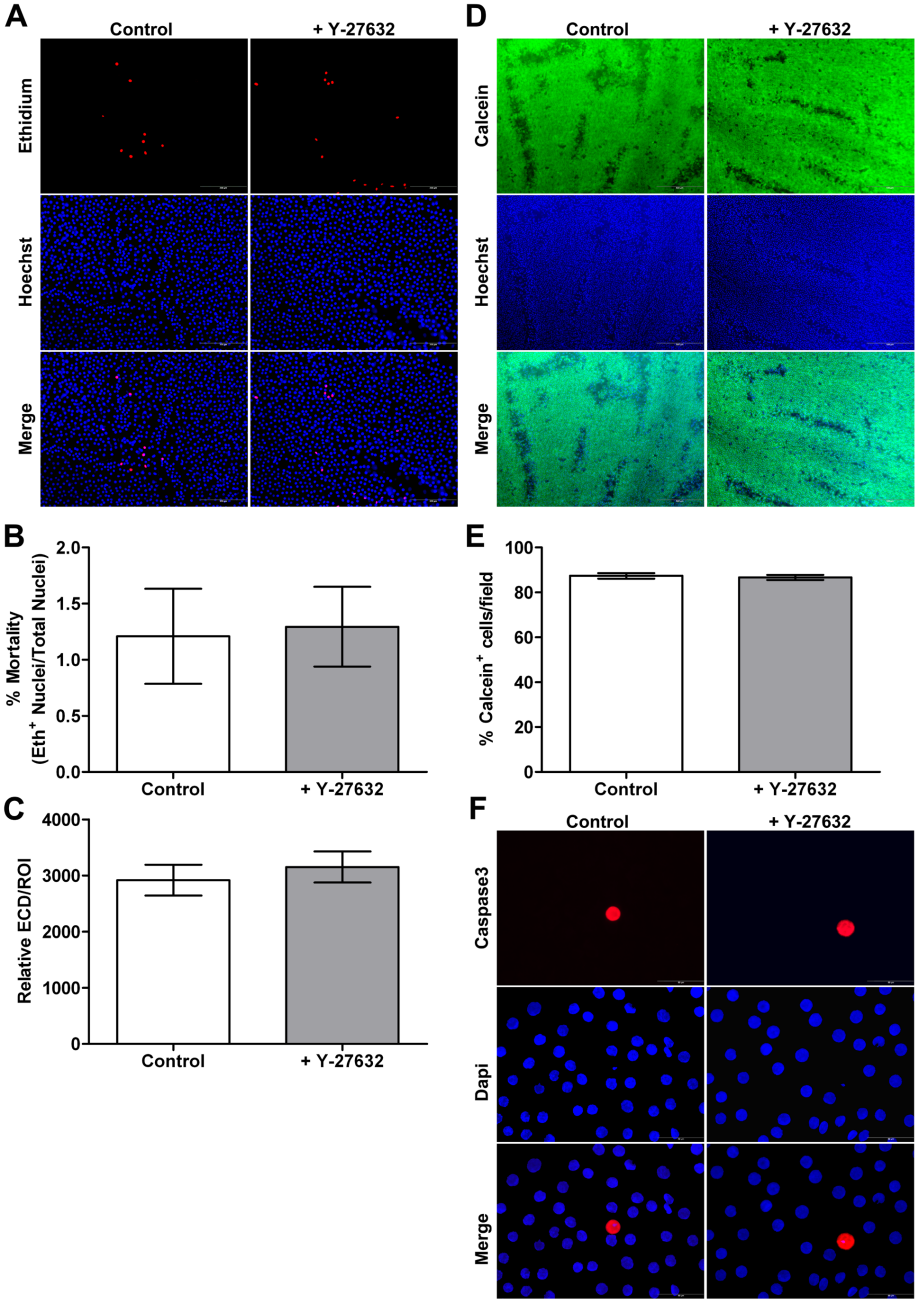
*Ex vivo* toxicity-viability and apoptosis assessment. (**A**) Toxicity evaluation by Ethidium Homodimer and Hoechst staining. Some dead cells are present in all conditions. (Magnification 10x.) (**B**) Mortality rate (%) corresponding to the number of positive Ethidium nuclei out of the total number of nuclei (Hoechst+Ethidium nuclei). No differences are observed between groups. (**C**) Relative Endothelial Cell Density (ECD) corresponding to the ratio between the number of nuclei and the area of the Region Of Interest (ROI). No differences are observed between groups. (**D**) Cell viability evaluation by Calcein AM and Hoechst staining. Magnification 4x. (**E**) Viability rate (%) corresponding to the ratio between the surface of positive Calcein cells and the field measurement area. No differences are observed between groups. (**F**) Apoptosis evaluation by Caspase3 immunostaining. Caspase3 positive cells were rarely observed. No differences are observed between groups. (Magnification 40x.) Values are means +/− SEM (n = 3 pairs). No differences are observed between groups.

The endothelium of control and treated corneas showed only a few red nuclei (Ethidium Homodimer positive HCEC) indicating dead cells (Figure 2A). Means mortality were not significantly different (P = 0.9) between both groups with respectively 1.2+/−0.4% and 1.3+/−0.4% of dead cells (Figure 2B). Relative ECD was also assessed in order to evaluate Y-27632 cytotoxicity. No statistically significant difference was observed between groups with 2919+/−274 and 3153+/−277 cells/ROI area (P = 0.36) for control and treated corneas respectively (Figure 2C). The endothelium of both groups showed also mainly green fluorescence (Calcein-AM), indicating live cells (Figure 2D). No statistically significant differences between control and treated corneas were observed (P = 0.6961) with respectively 87.4+/−1.3% and 86.7+/−1.2% of Calcein positive staining on the corneal surface (Figure 2E). Finally, the HCEC apoptosis status was examined by activated caspase3 immunostaining. Only few cells in both groups were caspase3 positive as shown in the Figure 2F. ROCK inhibitor had no acute cytotoxic and did not trigger apoptosis in HCEC.

### Ex vivo Proliferation Assessment

**Figure 3:**
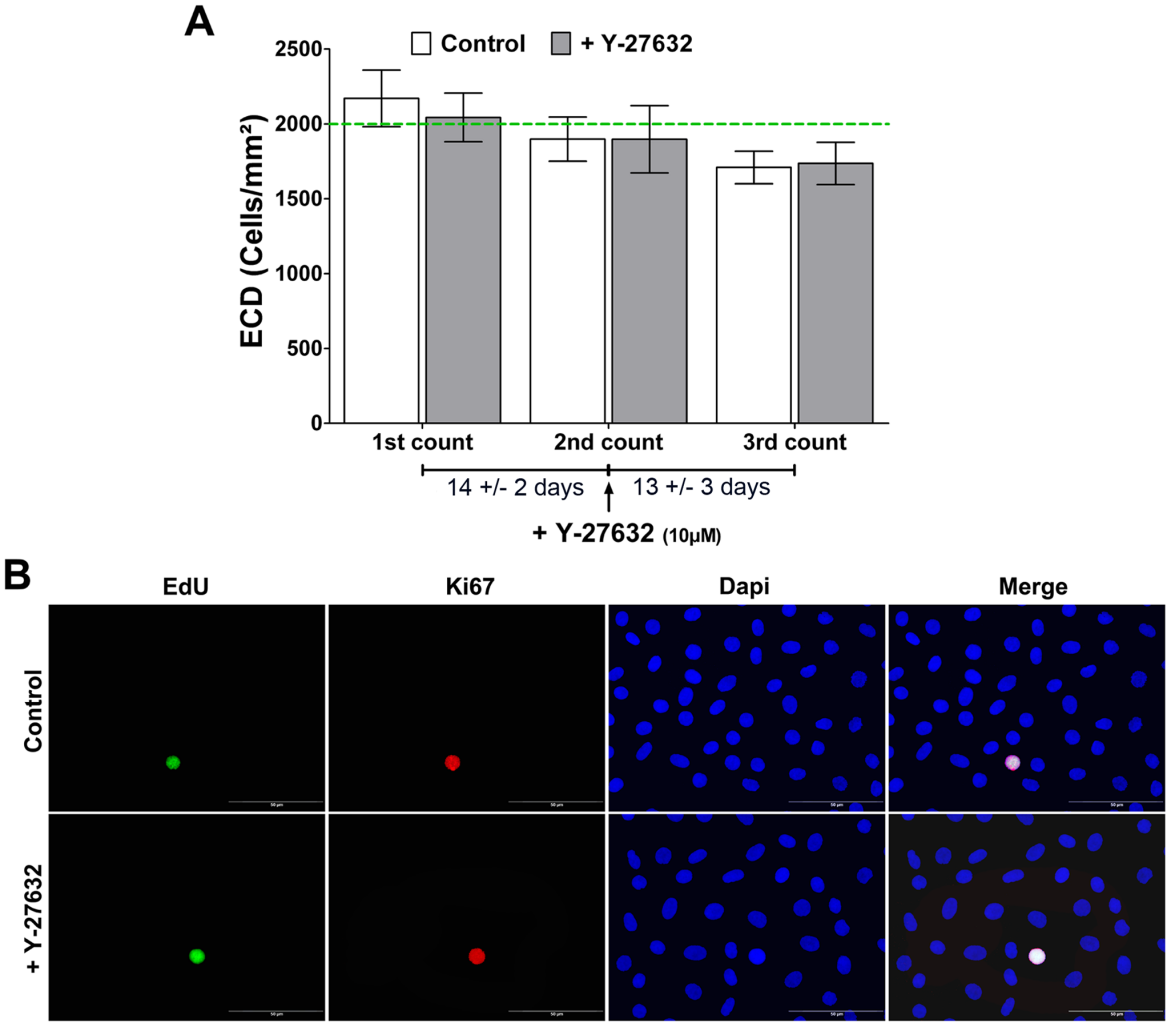
*Ex vivo* proliferation assessment. (**A**) Proliferation evaluation by Endothelial Cell Density (ECD) determination. ECD after corneas procurement is comparable in the control and the treated corneas. A cell loss of 12% and 9% for control and treated corneas respectively is observed after corneas storage during 14+/−2 days with no significant differences between both groups. A similar cell loss is observed in control and Y-27632 treated corneas after 13±3 days of preservation with respectively 8% and 9%. The threshold of 2000 cells/mm^2^ conventionally used to deliver grafts for penetrating keratoplasty is shown as a green dotted line. Values are means +/− SEM (n = 6 pairs). (**B**) Proliferation evaluation by EdU incorporation and Ki67 immunostaining. Only few EdU and Ki67 positive cells were detected in both groups. (Magnification 40x).

Effects of Y-27632 on *ex vivo* HCEC proliferation were firstly evaluated by routine endothelial cell count (Figure 3A). Endothelial baseline characteristics after corneas procurement were comparable, with 2171+/−189 in the control and 2043+/−162 cells/mm^2^ in the treated corneas (P = 0.62). After corneas preservation in OC medium during 14+/−2 days, ECD were 1899+/−148 *vs.* 1898+/−225 cells/mm^2^ (P = 0.99), corresponding to non-significant cell loss of 12% and 9% for control and treated corneas respectively. After Y-27632 treatment and OC preservation during 13+/−3 days, final ECD were 1709+/−109 *vs.* 1736+/−141 cells/mm2 (P = 0.88), corresponding to non-significant cell loss of 8% and 9% for control and treated corneas respectively. This ECD decrease in both groups before and after ROCK inhibitor treatment can be correlated to the mean cell loss generally observed during OC.

Effects of Y-27632 on *ex vivo* HCEC proliferation were also assessed by EdU incorporation and Ki67 immunostaining (Figure 3B). Few EdU and Ki67 positive cells were detected in control corneas confirming the low HCEC proliferative status *ex vivo*. No differences in the two proliferation markers staining between Y-27632 treated cells and controls could be observed, confirming ECD results. Endothelial cells proliferative capacities *ex vivo* are insufficient to compensate the daily cells loss, explaining the ECD decrease. ROCK inhibitor has no effect on the HCEC proliferative activity in OC corneas.

### Ex vivo Morphological Assessment

**Figure 4:**
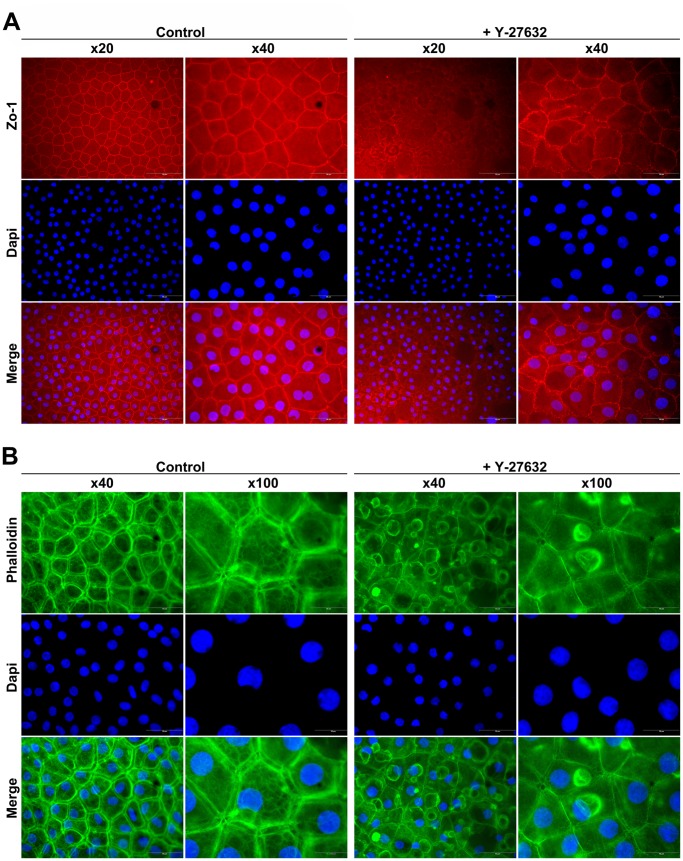
*Ex vivo* morphological assessment. (**A**) Zo-1 immunostaining. In control corneas, endothelial cells appear as a mosaic of cells with polygonal shape. Addition of Y-27632 induces a morphological change with a loss of the polygonal shape and an irregular cell border, suggesting a disruption of the tight junctions. (**B**) Actin staining. In control corneas, actin filaments are assembled into large radial and circumferential bundles, with a main localization along the membrane of the endothelial cells. After treatment the distribution of F-actin are altered, with only a residual staining associated with the cell periphery. The formation of circular membrane ruffles of variable size and actin content can also be observed.

The effect of ROCK inhibitor on the morphology of HCEC *ex vivo* was then examined. Without treatment, HCEC appeared as a mosaic of cells with polygonal shape. They were characterized by tight junctions surrounded the entire plasma membrane with regular expression of Zo-1, a member of a submembranous cytoplasmic complex associated with tight junctions. Addition of Y-27632 in the OC medium induced a morphological change with a loss of the polygonal shape and an irregular cell border, suggesting a disruption of the tight junctions as shown by the expression pattern of Zo-1 (Figure 4A).

In order to examine the cytoskeleton structure, phalloidin was used to investigate the distribution of actin filament in cells. In control corneas, actin filaments were assembled into large radial and circumferential bundles, with a main localization along the membrane of the endothelial cells. After treatment with ten µM Y-27632, the distribution of F-actin was dramatically altered, with only a residual staining associated with the cell periphery. The formation of circular membrane ruffles of variable size and actin content could also be observed in the endothelium of treated corneas (Figure 4B). Inhibition of Rho-ROCK pathway in corneal endothelium induces rearrangement of cytoskeleton.

### Ex vivo Wound Healing Assessment

**Figure 5:**
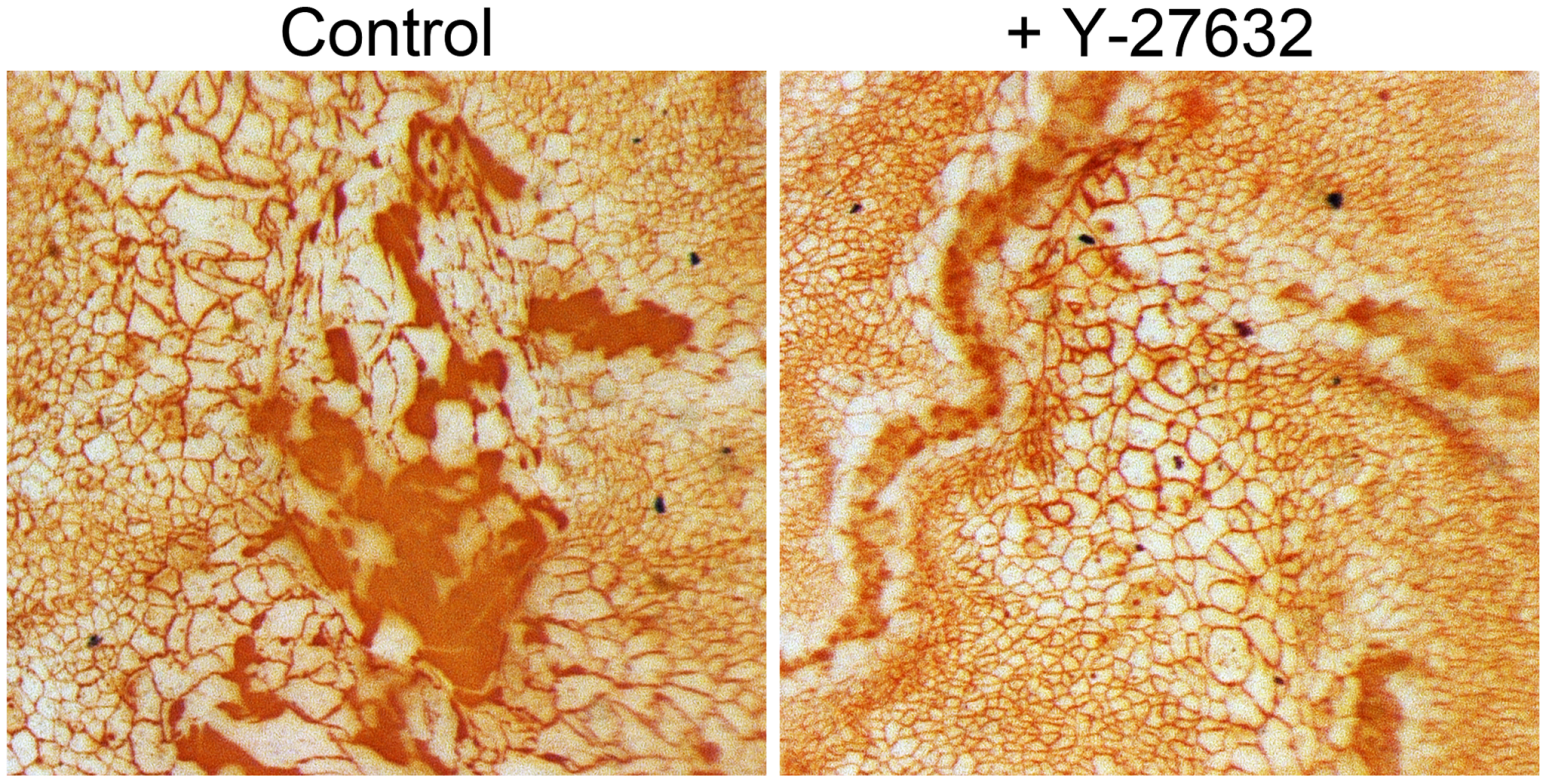
*Ex vivo* wound healing assessment. Y-27632 induces a totally wound closure after 12+/−4 days, while acellular areas could be still observed in the control group. Treated endothelial cells appear bigger than cells from unwounded region and have a regular shape. A pleomorphism is detected in the control group. (Magnification 60x).

Y-27632 was then evaluated as an enhancer of endothelial wound healing *ex vivo*. Treatment with ten µM Y-27632 induced a totally wound closure after 12+/−4 days, while acellular areas could be still observed in the control group (Figure 5). Even if, as previously described, EdU positive cells were detected in periphery of the wound [31], no proliferation differences could be seen between treated and control cornea (data not shown). These results demonstrated that ROCK inhibitor has enhanced endothelial wound healing *ex vivo*, likely due to increased migration and not proliferation.

### In vitro Toxicity, Viability and Apoptosis Assessment

**Figure 6:**
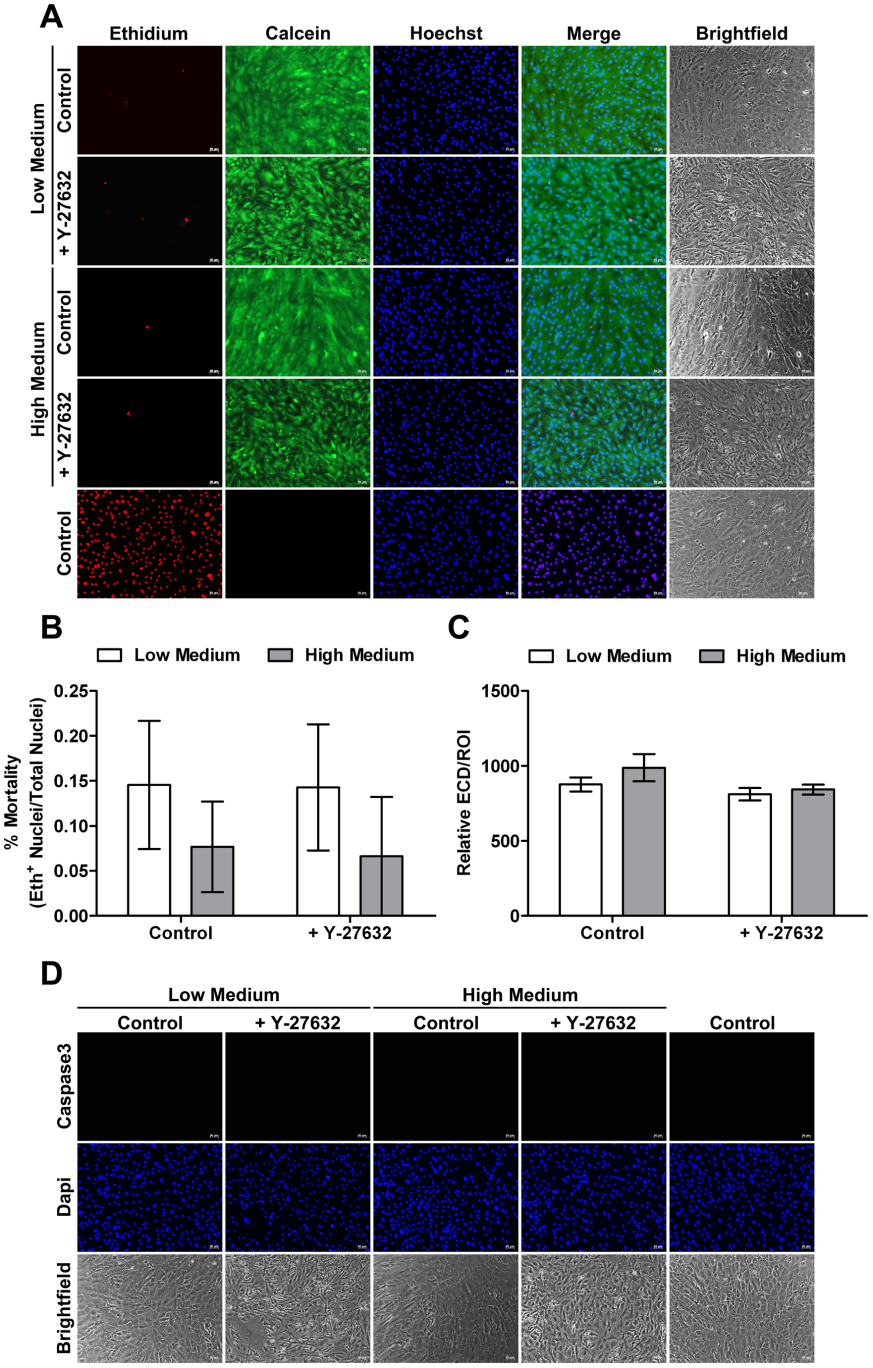
*In vitro* toxicity-viability and apoptosis assessment. (**A**) Toxicity and viability evaluation by triple staining Hoechst (nuclei) Ethidium (dead cells) and Calcein (viable cells). Methanol-fixed cultures act as positive controls for cell death. Few dead cells are present in all conditions and Calcein staining shows no differences in the cell viability after Y-27632 treatment. (**B**) Mortality rate (%) corresponding to the number of positive Ethidium nuclei out of the total number of nuclei (Hoechst+Ethidium nuclei). No differences are observed between all groups. (**C**) Relative Endothelial Cell Density (ECD) corresponding to the ratio between the number of nuclei and the Region Of Interest (ROI) measurement area. No differences are observed between all groups. (**D**) Apoptosis evaluation by Caspase3 immunostaining. No staining is observed in all groups. (Magnification 10x). Values are means +/− SEM (n = 9).

Representative micrographs of the cell-viability assay are presented in Figure 6A. For this assay, methanol-fixed cultures acted as positive controls for cell death. Fluorescence microscopy of methanol-fixed cultures revealed red-stained nuclei throughout the culture. A small percentage of dead cells were observed in confluent primary cultures in the different incubation conditions (Figure 6B). As shown in Table 1 no statistically significant differences in the cell mortality were observed between all groups. Such as in the *ex vivo* study, relative ECD was assessed in order to evaluate Y-27632 cytotoxicity (Figure 6C). Again, no statistically significant differences were observed between the different groups (Table 1). Furthermore, Calcein staining showed no differences in the cell viability after Y-27632 addition in Low or High Medium culture indicating that all cells conserved esterase activity (Figure 6A). In order to evaluate the apoptotic action of ROCK inhibitor on endothelial cell culture, caspase3 immunostaining was performed. Regardless of the incubation conditions no caspase3 positive cells were observed (Figure 6D). Similar results were obtained on non-confluent primary cell culture (data not shown). These results demonstrated that, like in *ex vivo* study, Y-27632 had no acute cytotoxic on HCEC.

**Table 1:** Toxicity assessment on confluent primary cell culture.

Mortality (%)	Low medium control	High medium control	Low medium+Y-27632	High medium+Y-27632
**Mean+/−SEM**	0.14+/−0.07	0.08+/−0.05	0.14+/−0.07])	0.07+/−0.07
**Low medium control** **(P value)**		0.44	0.98	0.43
**High medium control** **(P value)**			0.46	0.90
**Low medium+Y-27632** **(P value)**				0.44
**Relative ECD (%)**	**Low medium control**	**High medium control**	**Low medium+Y-27632**	**High medium+Y-27632**
**Mean+/−SEM**	876+/−47	987+/−91	811+/−42	842+/−33
**Low medium control** **(P value)**		0.60	0.39	0.67
**High medium control** **(P value)**			0.14	0.30
**Low medium+Y-27632** **(P value)**				0.30

### In vitro Proliferation Assessment

**Figure 7:**
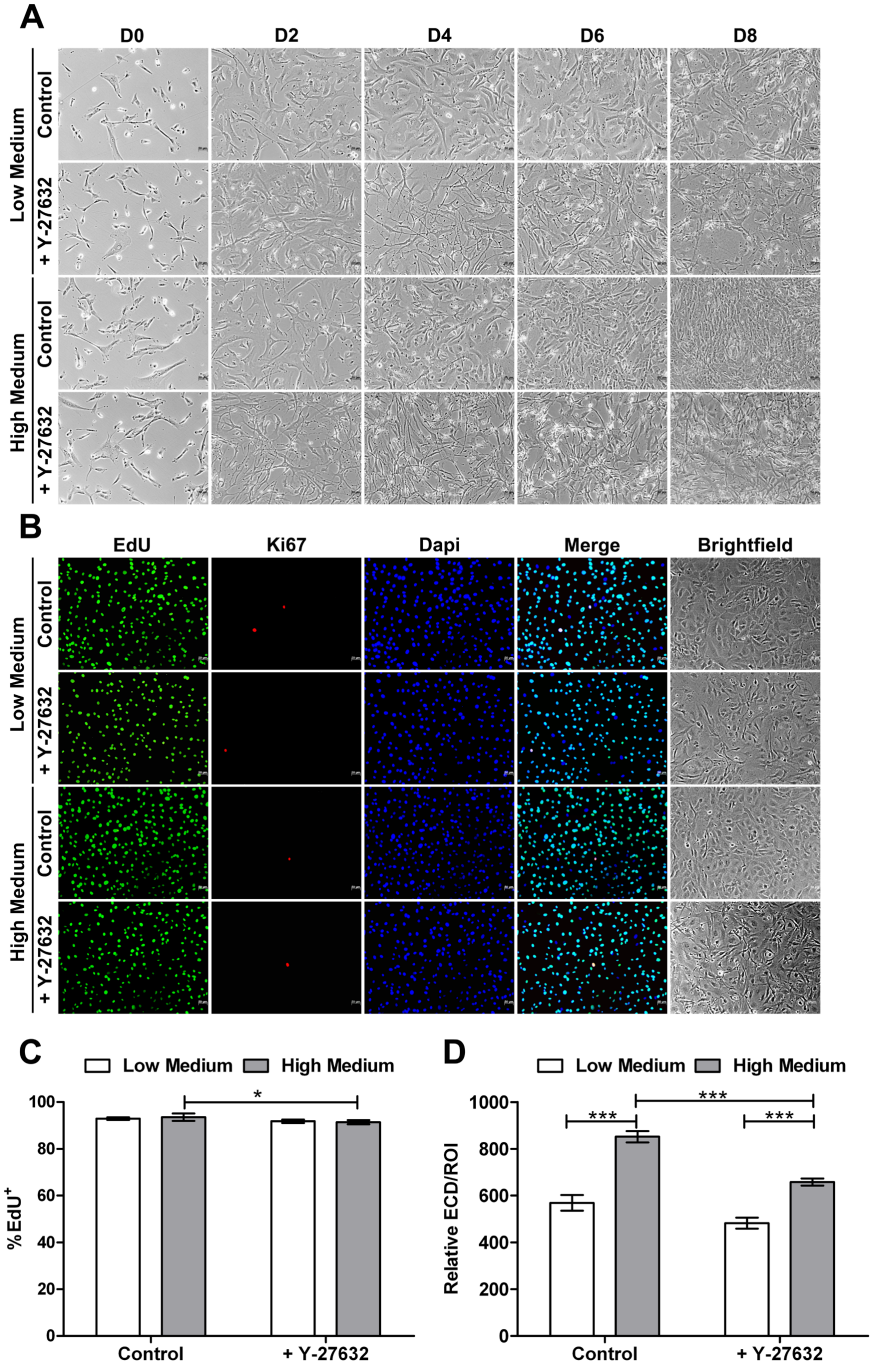
*In vitro* proliferation assessment. (**A**) Cell growth kinetic in Low or High Medium supplemented or not with Y-27632. Cells reach confluency more quickly in High Medium than in Low Medium. Cell shape in the two medium is different, with bigger cells and a more regular endothelial mosaic in Low Medium than in the High Medium where a poly and pleiomorphism are detected. Y-27632 addition induces modifications in cell proliferative potential and morphology. In both mediums, cells adopt a fibroblastic-like appearance. Additional time is necessary for cells to reach the confluency in the presence of Y-27632 whatever the medium. (**B**) Proliferation evaluation by EdU incorporation and Ki67 immunostaining. EdU and few Ki67 positive cells are detected in both groups. Magnification 10x. (**C**) Proliferation rate (%) corresponding to the number of positive EdU nuclei out of the total number of nuclei (EdU+Dapi nuclei). Globally no differences are noted between all groups, a light statistically significant decreased in EdU positive cells is observed between control and treated cells incubated in High Medium. (**D**) ECD in High Medium is higher than in Low Medium condition as well in the untreated as treated groups. A decreased of the ECD is observed in cells incubated in Low (−15%) and High Medium (−22%) supplemented with Y-27632 compared to the controls. Values are means +/− SEM (n = 9). *P<0.05 and ***<0.0001 (Student’s *t*-test).

Proliferation kinetic was evaluated by cell density and EdU incorporation every day until cells reached confluency with or without Y-27632 addition in Low and High Medium. A difference in cell proliferation could be observed between the different groups. As shown in Figure 7A, cells reached confluency more quickly in High Medium than in Low Medium demonstrating, as expected, that High Medium is better to expand HCEC. Furthermore, cell shape in the two mediums were different, with bigger cells and a more regular endothelial mosaic in Low Medium, comparable to the *in vivo* structure, than in the High Medium where a pleomorphism was detected. The addition of Y-27632 induced also some modifications in cell proliferative potential and morphology. In both mediums, cells adopted a fibroblastic-like appearance suggesting a higher capacity to migrate than to proliferate. Indeed additional time was necessary for cells to reach the confluency in the presence of Y-27632 whatever the medium, demonstrating a reduction of the HCEC proliferative capacity. These results were also evaluated by Ki67 immunostaining and EdU incorporation assay on HCEC at confluency (Figure 7B). Only few Ki67 positive cells were detected in all conditions, which showed a low cell proliferative capacity of HCEC at confluency. Even if, as presented in Figure 7C, globally no differences were noted between all groups, a statistically significant, but low decrease in EdU positive cells was observed between control and treated cells incubated in High Medium with respectively 93.6+/−1.6% and 91.4+/−0.9% of EdU positive cells (P = 0.02). Relative ECD was also assessed to estimate the effect of ROCK inhibitor on the HCEC proliferative ability (Figure 7D). ECD in High Medium condition was higher than in Low Medium, either untreated or treated, with respectively 852+/−24 *vs*. 569+/−33 cells/ROI area in control culture (P<0.0001) and 658+/−15 *vs*. 482+/−23 cells/ROI area in treated culture (P<0.0001). In addition, a significant decreased of the ECD (-22%) was noted in cells incubated in High Medium supplemented with Y-27632 compared to the control (P<0.0001). A similar, but not significant, decrease of the ECD (-15%) was observed in Low Medium culture with ROCK inhibitor compared to Low Medium control condition. These results indicate that High Medium was better than Low Medium for HCEC cultivation and inhibition of Rho-ROCK pathway reduced cell proliferation capacity of HCEC *in vitro*.

### In vitro Morphological Assessment

**Figure 8:**
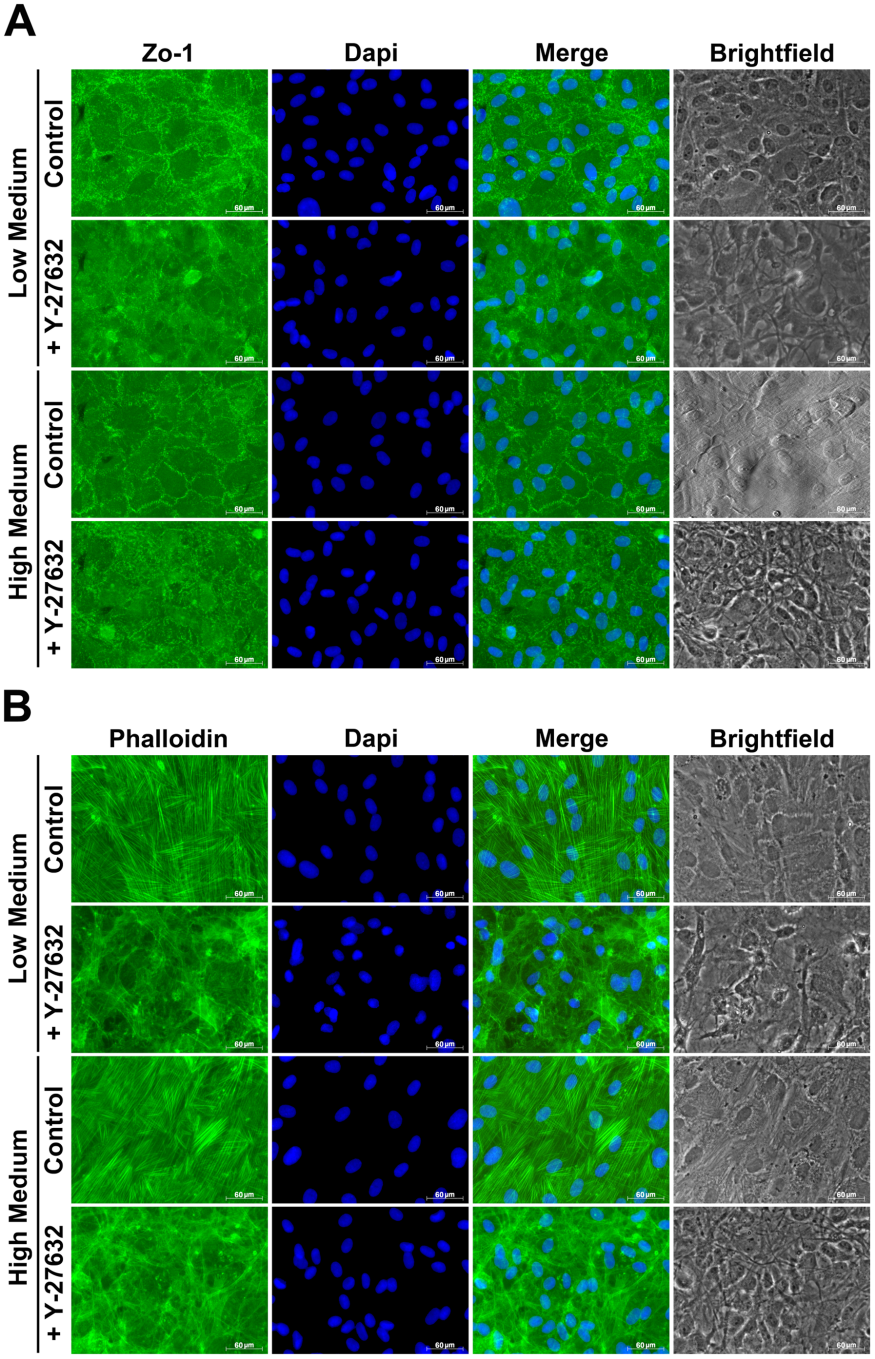
*In vitro* morphological assessment. (**A**) Zo-1 immunostaining reveals a continuous or partially segmented expression at intercellular junction in both conditions. This expression pattern is completely lost after Y-27632 addition in Low or High Medium culture with more diffuse intracellular staining. (**B**) Actin staining reveals radial and circumferential actin bundles in control groups. Y-27632 induces cytoskeleton reorganization, characterized by a redistribution of actin microfilament on the periphery of the cells and apparition of some membrane ruffles. (Magnification 40x) (n = 9).

As for *ex vivo* experiment, the effect of ROCK inhibitor on HCEC was examined *in vitro*. In untreated culture, Zo-1 immunostaining revealed a continuous or partially segmented expression at intercellular junction in both conditions. This expression pattern was completely lost after Y-27632 addition in Low or High Medium culture with more diffuse intracellular staining, suggesting a disruption of the tight junctions as already seen in OC corneas (Figure 8A). To evaluate whether the actin structure was also affected *in vitro*, phalloidin staining was performed in the different conditions of culture. Without Y-27632, the distribution of actin was mainly the same as for *ex vivo* cornea, with radial and circumferential actin bundles. Treatment with Y-27632 induced cytoskeleton reorganization, characterized by a redistribution of actin microfilament on the periphery of the cells and apparition of some membrane ruffles. This redistribution of actin was seen in High and Low medium (Figure 8B).

### In vitro Wound Healing Assessment

**Figure 9:**
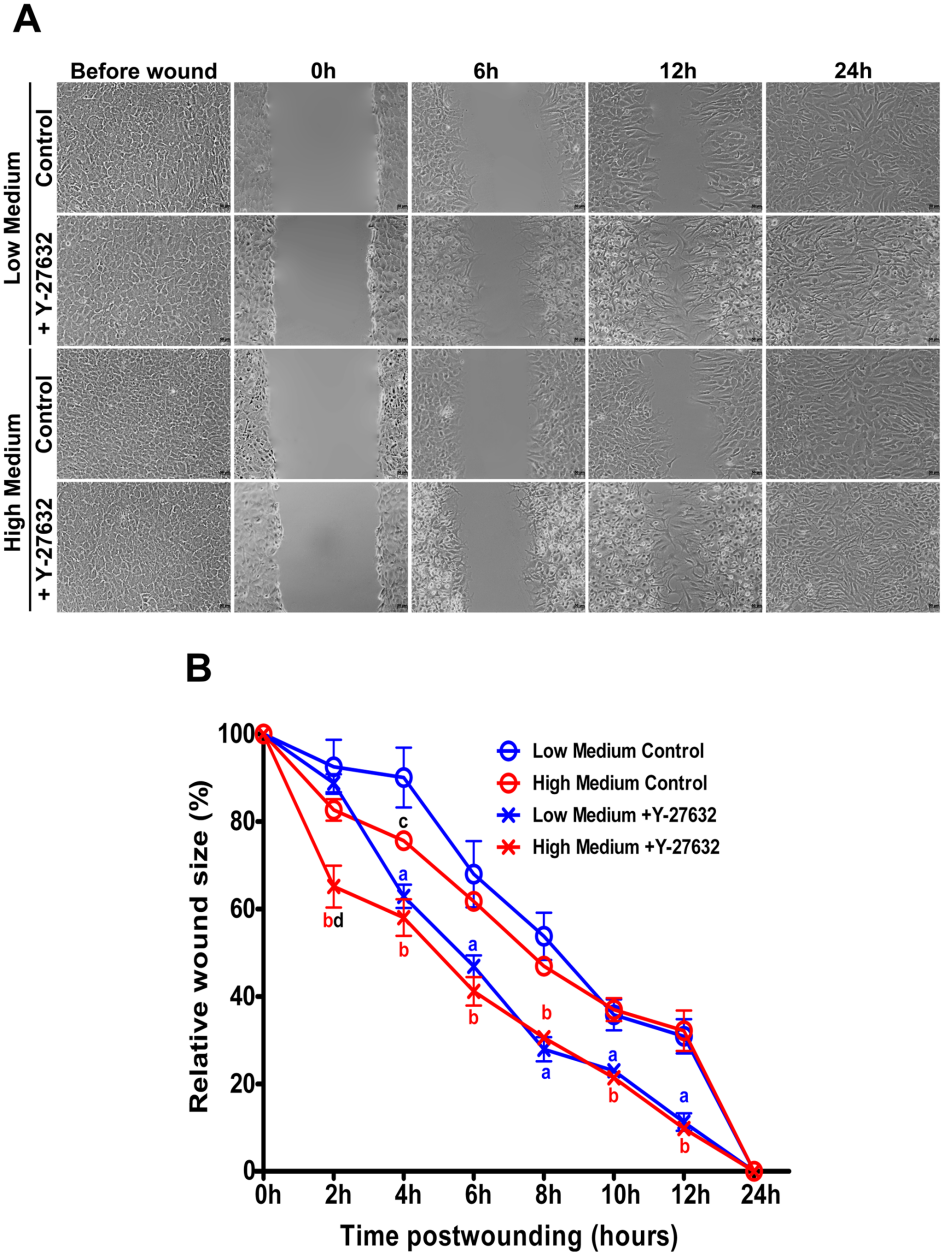
*In vitro* wound healing kinetic. (**A**) Representative micrographs of the wound healing kinetic. Wounds are totally closed whatever the conditions incubation after 24 hours. Compared to controls, Y-27632 enhances endothelial wound closure and cells adopt a fibroblastic-like appearance 6 hours after treatment. Magnification 10x. (**B**) Relative wound closure kinetic. Y-27632 significantly increases endothelial wound closure compared to controls (P = 0.0004). Globally no differences are observed between the Low and the High Medium in the wound healing process. a-d: P<0.05; a: Low Medium Control vs. Low Medium +Y-27632; b: High Medium Control vs. High Medium +Y-27632; c: Low Medium Control vs. High Medium Control; d: Low Medium +Y-27632 vs. High Medium +Y-27632 (2way Anova test; n = 9).

**Figure 10:**
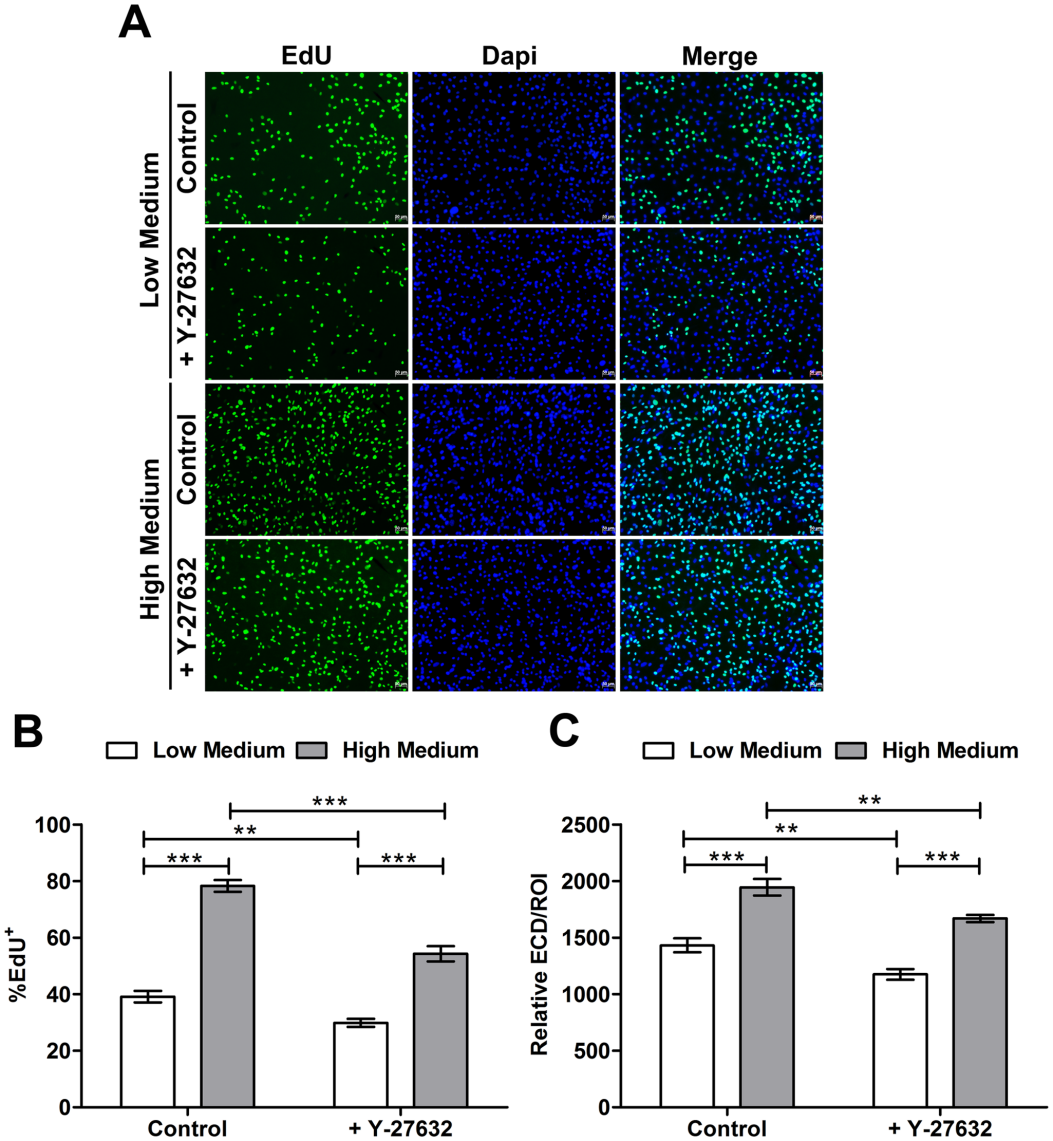
*In vitro* proliferation assessment during wound healing. (**A**) EdU incorporation evaluation during wound healing process. EdU staining can be observed in all conditions. Magnification 10x. (**B**) Proliferation rate (%) corresponding to the number of positive EdU nuclei out of the total number of nuclei (EdU+Dapi nuclei). High Medium increases EdU incorporation compared with Low Medium in control cultures but also in Y-27632 treated cultures during wound healing process. Furthermore, Y-27632 treatment decreases the number of EdU positive cells compared to control groups with a decrease of 23.6 and 30.7% in Low and High Medium respectively. (**C**) Relative ECD corresponding to the ratio between the number of nuclei and the ROI measurement area. Cells incubated in High Medium have a superior Relative ECD in control and treated group compared with cells incubated in Low Medium of respectively 26.4% and 29.6%. Y-27632 treatment decreases the Relative ECD of 17.9% in Low Medium and of 14.2% in High medium compared to controls. Values are means +/− SEM (n = 9). **P<0.001 and ***P<0.0001 (Student’s *t*-test).

To determine the role of Rho pathway in endothelial wound healing, we assessed the effects of Y-27632 on endothelial wound closure of HCEC monolayer. Confluent culture cells were scraped with a pipet tip to create cell-free wounds. Cells were then incubated in Low or High Medium supplemented or not with Y-27632. Wound healing was observed every 2 hours until 12 hours and after 24 hours post-wound. Representative micrographs of the wound healing kinetic are presented in Figure 9A. Compared to controls, Y-27632 significantly enhanced endothelial wound closure (P<0.001). Globally no differences were observed between the Low and the High Medium in the wound healing process. Wounds were totally closed whatever the conditions after 24 hours (Figure 9B). To confirm the cell migration involvement in wound closure and not the cell proliferation, EdU was added at the same time than Y-27632 and revealed after 24 hours when wounds were totally closed (Figure 10A). As presented in Figure 10B and Table 2, large differences can be observed between groups. High Medium increased EdU incorporation compared with Low Medium (P<0.0001) in control cultures but also in Y-27632 treated cultures (P<0.0001) during wound healing process. Furthermore, Y-27632 treatment decreased the number of EdU positive cells compared to control groups with a decrease of 23.6 and 30.7% in Low and High Medium respectively (P<0.0001). Relative ECD was also assessed in order to indirectly evaluate the involvement of Y-27632 on cell proliferation during the wound healing *in vitro* (Figure 10C and Table 2). An effect of the medium could be observed on the relative ECD in the control and treated group. Cells incubated in High Medium had a superior Relative ECD in control and treated group compared with cells incubated in Low Medium of respectively 26.4% and 29.6%. Y-27632 treatment decreased the Relative ECD of 17.9% in Low Medium and of 14.2% in High medium compared to controls. These results demonstrated that ROCK inhibitor promotes corneal endothelial wound healing *in vitro* by inducing cell motility and not cell proliferation.

**Table 2:** % EdU positive cells and relative ECD after wound healing in vitro.

EdU (%)	Low medium control	High medium control	Low medium+Y-27632	High medium+Y-27632
**Mean+/−SEM**	39.09+/−2.07	78.33+/−2.09	29.85+/−1.46	54.29+/−2.73
**Low medium control** **(P value)**		*<0.0001*	*0.0021*	
**High medium control** **(P value)**				*<0.0001*
**Low medium+Y-27632** **(P value)**				*<0.0001*
**Relative ECD (%)**	**Low medium control**	**High medium control**	**Low medium+Y-27632**	**High medium+Y-27632**
**Mean+/−SEM**	1432+/−61	1945+/−72	1175+/−48	1669+/−31
**Low medium control** **(P value)**		*<0.0001*	*0.0043*	
**High medium control** **(P value)**				*0.0029*
**Low medium+Y-27632** **(P value)**				*<0.0001*

### In vitro Cell Adhesion Assay

**Figure 11:**
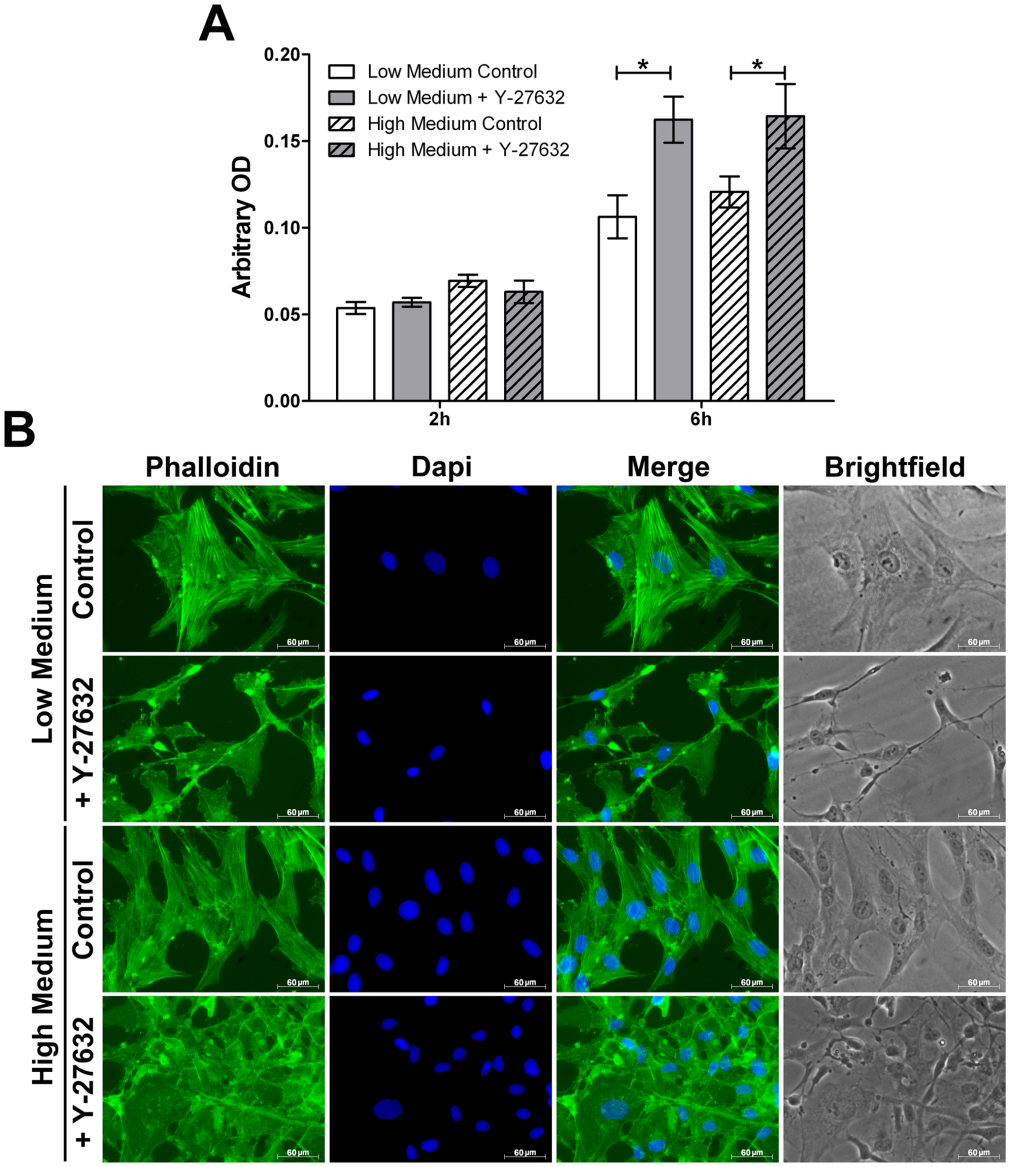
*In vitro* cell adhesion assay. (**A**) No differences in cell adhesion are observed between the different groups after 2 hours of incubation. After an incubation of 6 hours a higher number of HCEC adheres onto fibronectin matrix compared with lower incubation time. Y-27632 enhances cells adhesion after 6 hours compared to control medium. Medium (Low or High) is not involved in cell adhesion process. Values are means +/− SEM (n = 9). *P<0.05 (Student’s *t*-test). (**B**) Micrographs of Phalloidin staining. An actin remodeling after ROCK inhibitor treatment can be observed. (Magnification 40x) (n = 9).

We next determined whether Y-27632 might play a role in cell adhesion in HCEC. As shown in Figure 11A, no differences were observed between the different groups after 2 hours of incubation. After 6 hours a significantly higher number of HCEC adhered onto fibronectin matrix compared with lower incubation time (P<0.0001). Treatment of HCEC with Y-27632 enhanced in a significant manner cells adhesion after 6 hours compared to control medium (P<0.05). It can be also noted that medium (Low or High) was not involved in cell adhesion process (P>0.05). As already described above, phalloidin staining showed actin remodelling after ROCK inhibitor treatment, suggesting that adhesion enhancement could be due to cytoskeleton reorganization through Rho signalling pathway (Figure 11B).

## Discussion

Loss of visual acuity, following corneal endothelial dysfunction, is one of the major indications for corneal transplantation. Corneal grafts are retrieved and evaluated by eye bank before to be use in clinics and the main criteria for clinical eligibility of cornea is the ECD. It is representative of the functional capacity of the endothelium and a minimal density of 2000 cells/mm^2^ is required for corneal transplantation. Due to the incapacity of HCEC to proliferate *ex vivo*, loss of HCEC during OC is the main reason for corneal rejection by tissue banks. This ECD decrease seems to be principally due to apoptosis during storage process [32]. Due to the shortage of donor corneas available for transplantation, several groups tried to limit this ECD decrease, either by restraining the cell loss or by inducing proliferation of the endothelial cell layer. Using animal models either *in vivo* or *in vitro*, Kinoshita and colleagues have evaluated the role of ROCK inhibitor in CEC. They have shown that treatment of cynomolgus monkey cultivated CEC with selective ROCK inhibitor Y-27632 inhibited apoptosis and promotes proliferation [23]. These data suggest that ROCK inhibitor is able to modulate apoptosis and proliferation of monkey corneal endothelial cells *in vitro*. If this is also true in human, this inhibitor could be a potential pharmacological compound in order to optimize eye banking system. As one of the main goals of eye bank is to avoid the decrease of ECD during storage, addition of ROCK inhibitor could allow limiting the cell loss by its anti-apoptotic activity and/or increasing ECD by enhancement of cell proliferation.

In the present study, we first evaluated the toxicity of Y-27632 *ex vivo*. We demonstrated that this compound had no toxicity effect and did not modulate viability of HCEC, suggesting that this molecule is safe to be used in eye bank or in clinic. However, in contrast to a previous report on animal models [23], ROCK inhibitor treatment was not able to induce proliferation or to reduce apoptosis *ex vivo*, as shown by EdU incorporation, as demonstrated by the ECD loss observed during storage and by Caspase3 immunostainig. HCEC has been shown to possess proliferative capacity, but *in vivo* conditions seem to contribute to maintenance of a non-replicative monolayer. Several factors are involved in these antiproliferative mechanisms, including TGF beta 2 in aqueous humor and a high contact inhibition present in the corneal endothelial mosaic mediated by the cyclin kinase inhibitor p27Kip1 [33]. In absence of these factors, HCEC can be induced to growth in culture. This first result demonstrated that treatment with Y-27632 was not strong enough to induce proliferation and to overcome these antiproliferative mechanisms induced by contact inhibition *ex vivo*. As ROCK inhibitor has been shown to induce proliferation of rabbit and monkey CEC *in vitro*, we also evaluated the effect of Y-27632 in human primary cell culture. As per *ex vivo* evaluation, treatment with ROCK inhibitor did not show any toxicity on HCEC, demonstrating a potential safe use of this compound for cell culture. However, inhibition of Rho-ROCK pathway did not induce proliferation of HCEC as it was the case for rabbit and monkey endothelial cells, but actually reduced cell proliferation capacity of HCEC *in vitro.* These findings rather confirmed previous studies demonstrating that inhibition of ROCK signaling induced a reduction of proliferation of different cell type, including corneal epithelial cells [30], vascular smooth muscle cell [34], cardiomyocytes [35] and myofibroblast [36]. These first results demonstrated that ROCK inhibitor, although non-toxic for the HCEC, will not be the key factor which allows a greater number of human corneal grafts to become available clinically or which promotes cultivation of HCEC.

This unpredicted difference in induction of proliferation could be explained by the effect of donor age on HCEC proliferative capacity. It has been shown that cultivated HCEC from older donors had lower proliferative potential than those derived from younger donors. This lower potential seems to be due to the significant increase with age of two cyclin kinase inhibitors, p21Cip1 and p16INK4, which are important negative regulator of G1-phase of the cell cycle [37]. In our study the mean donor’s age was 73 years old and could be considered as old donors, whereas animals used for the experiment are generally young and would rather correspond to young donors. Even if younger samples will be difficult to obtain (average donor age in the eye bank in Lausanne was 69 years old in 2010), it would be interesting to evaluate the effect of ROCK inhibitor on HCEC coming from young donors *ex vivo* and *in vitro*. This study will allow evaluating definitively, whether there is a difference between the action of Y-27632 in young and old HCEC and so whether ROCK inhibitor could have an effect on the proliferation induction of some young populations of HCEC.

Variation has been also observed between species related to proliferative capacity. Bovine, rabbit and rat endothelial cells grow easily in culture, whereas monkey and human do not. Culture rabbit and human cells have been used to compare corneal endothelial cell cycle and differential expression of cell cycle-related proteins has been evaluated. The only observed difference is the localization of cyclin E, which is located in the cytoplasm of rabbit cells and in the nucleus of human cells [38]. Furthermore, another study has shown that FGF-2-mediated cell proliferation is differentially regulated in rabbit and human corneal endothelial cells. Even if the principal step is mediated by phosphorylation and degradation of p27kip1 in both species, this induction of proliferation involved PI3-kinase-dependent ERK1/2 activation in human, while this effect is induced by these two pathways in parallel and independently in rabbit [39]. These results suggest that cells derived from rabbit and human are arrested and/or regulated at different point within G1-phase. It could be possible that the action of the ROCK inhibitor related to the activation of the cell cycle is different in human and in animal models, explaining why such a difference is observed in term of induction of proliferation.

Besides this induction of proliferation, Kinoshita and colleagues have demonstrated that ROCK inhibitor promoted in vitro wound healing of cultivated monkey CEC and administrated as an eye drop, enhanced corneal endothelial wound healing in a rabbit model, damaged by transcorneal freezing [24]. The same group has also observed that injection of cultivated CEC treated with ROCK inhibitor enables regeneration of cornea in a rabbit or monkey corneal endothelial dysfunction model. Even if a previous study reported that cell injection in anterior chamber was ineffective, as the cells appears to be wash off by aqueous humor flow [40], they suggested that the injection of cultivated HCEC in presence of Y-27632 could be a potential therapeutic strategy in order to cure corneal endothelial dysfunction [25]. Although promising in animal models, the effect of ROCK inhibitor on wound healing and adhesion was never tested in human endothelial cornea. Evaluation of this compound in human cornea will be an essential step before clinical application. In our study, we confirmed that ROCK inhibitor is able to enhance adhesion and wound healing on human corneal endothelial cells. Furthermore, we demonstrated that inhibition of ROCK signaling enhanced endothelial wound closure in a proliferation-independent manner, confirming previous results of this study and strongly suggesting that cell migration primarily accounted for the observed effect. Migration process involved membrane protrusion through cytoskeleton modification and establishment of new adhesion sites with the substratum over which it migrated. Our study revealed that inhibition of ROCK signaling induced a morphological change of HCEC, characterized by a loss of the polygonal shape and a remodeling of the cytoskeleton, as shown by the redistribution of actin on the periphery of the cells and the formation of circular membrane ruffles. The ability of ROCK to control cells migration and adhesion of corneal endothelial cells seems to be related to its role in the regulation of the dynamic rearrangements of the actin cytoskeleton. Further investigations should be performed to better understand the mechanisms involved in the enhancement of wound healing and adhesion by ROCK inhibitor.

In summary, ROCK inhibitor did not shown any toxicity, but is not the key compound allowing inducing proliferation or limiting apoptosis of HCEC in eye banking culture system. ROCK signaling negatively regulates adhesion and wound healing in human corneal endothelial cells, via modulating the cytoskeleton. Our results strongly suggest that ROCK inhibitor could be used in clinic for corneal endothelium dysfunction, either as a direct treatment for wound healing with eye drop or for cell transplantation by modulation of the cell adhesion properties.
